# A systematic analysis of the expression of the anti-HIV VRC01 antibody in *Pichia pastoris* through signal peptide optimization

**DOI:** 10.1016/j.pep.2018.03.013

**Published:** 2018-09

**Authors:** Rochelle Aw, Paul F. McKay, Robin J. Shattock, Karen M. Polizzi

**Affiliations:** a Department of Life Sciences, Imperial College London, London, SW7 2AZ, UK; b Centre for Synthetic Biology and Innovation, Imperial College London, SW7 2AZ, UK; c Department of Infectious Diseases, Imperial College London, London, W2 1PG, UK

**Keywords:** *Pichia pastoris / Komagataella phaffi*, Broadly neutralizing antibody, VRC01, Signal peptide, 2A sequence, AA, α-amylase, α-MF, α-mating factor, αK, α-mating factor K, αKS, α-mating factor KS, αT, α-mating factor T, AOX1, alcohol oxidase 1, AP, alkaline phosphate, BMGY, buffered glycerol complex medium, BMMY, buffered methanol complex medium, bNAbs, broadly neutralizing antibodies, clonNAT, Nourseothricin, GA, glucoamylase, HEK, human embryonic kidney, IgG, Immunoglobulin G, IN, inulinase, IV, invertase, KP, killer protein, LZ, lysozyme, SA, serum albumin, YPD, yeast peptone dextrose

## Abstract

*Pichia pastoris* (*Komagataella phaffi)* has been used for recombinant protein production for over 30 years with over 5000 proteins reported to date. However, yields of antibody are generally low. We have evaluated the effect of secretion signal peptides on the production of a broadly neutralizing antibody (VRC01) to increase yield. Eleven different signal peptides, including the murine IgG1 signal peptide, were combinatorially evaluated for their effect on antibody titer. Strains using different combinations of signal peptides were identified that secreted approximately 2–7 fold higher levels of VRC01 than the previous best secretor, with the highest yield of 6.50 mg L^−1^ in shake flask expression. Interestingly it was determined that the highest yields were achieved when the murine IgG1 signal peptide was fused to the light chain, with several different signal peptides leading to high yield when fused to the heavy chain. Finally, we have evaluated the effect of using a 2A signal peptide to create a bicistronic vector in the attempt to reduce burden and increase transformation efficiency, but found it to give reduced yields compared to using two independent vectors.

## Highlights

• Production of VRC01 antibody in *Pichia pastoris* was increased 2–7 fold.
• Using the murine IgG1 signal peptide on the light chain results in higher yields.
• The use of bicistronic vectors did not increase the yield of VRC01.

## 1 Introduction

The broadly neutralizing antibody (bNAb) VRC01 is currently undergoing Phase II clinical trials to determine its effectiveness in preventing HIV infection in high risk women and in men and transgender people who have sex with men [1,2]. VRC01 works by binding to the gp120 envelope glycoprotein and blocking interaction with the CD4^+^ receptor; thus, preventing viral entry into the T-cell [3]. *In vitro* VRC01 has been shown to neutralize 91% of HIV isolates, and therefore is a promising candidate in the fight against HIV [4]. Results from two initial Phase I clinical trials indicate that VRC01 has been identified as a being able to delay the return of viral replication following interruption of antiretroviral therapy [5].

In most cases VRC01 is produced in human embryonic kidney (HEK) cells, although production in plants has also been reported [6]. Yields for plant production were up to 100 mg/kg in stably expressing lines. We have previously reported on the production of the bNAb VRC01 using *Pichia* (syn. *Komagataella) pastoris*, with yields up to 3.05 mg L^−1^ in shake flasks [7]. Our research utilized the murine IgG1 signal peptide (GWSCIILFLVATATGVHSQ) for the first time in *P. pastoris*, which outperformed the more popular α-mating factor from *Saccharomyces cerevisiae* [8,9].

*P. pastoris* is often chosen as an expression host due to the high cell densities that can be achieved, up to 130 g L^−1^ dry cell weight, and therefore the corresponding high volumetric productivities [10]. *P. pastoris* secretes very few native proteins, thus purification can be made easier by directing recombinant proteins to the supernatant through the use of a signal peptide. There are many reported instances where the choice of signal peptides impacts the yields achieved for expression; for instance He et al. found that utilizing a bovine-casein signal peptide for the secretion of the xlyanase xynB resulted in lower yields than using the α-mating factor (α-MF) pre-pro peptide from *S. cerevisiae* [11]. Gasser et al. were unable to secrete antibody fragments using a mAb leader signal peptide, but were able to secrete the same products using the α-MF [12]. Conversely the expression of bovine pancreatic trypsin inhibitor (BPTI) was unsuccessful when using the α-MF, but utilizing the human serum albumin (HSA) signal peptide resulted in successful secretion into the medium [13].

There are various truncations to the α-MF signal peptides that have been demonstrated to increase the cleavage efficiency of the STE13 protease for some proteins [[14], [15], [16]]. In addition, a series of other signal peptides have in various forms successfully resulted in the production of recombinant proteins; α-amylase [17], glucoamylase [18], inulinase [19] invertase [20], killer protein [19], lysozyme [21], and human serum albumin [13,22]. As the optimal choice of signal peptides is often protein specific, testing different signal peptides should influence overall yield.

Another method to improve yield has been by using bicistronic vectors, which in the case of antibodies results in the production of both the heavy and the light chain from a single vector. These can be generated using internal ribosome entry sites (IRES) [23] or through the use of 2A peptide sequences [24]. Previously antibody fragments have been expressed using two expression cassettes in tandem; however, this requires two promoter and transcription terminator regions [12]. The IRES and 2A both utilise a single promoter, reducing the size of the vector and increasing transformation efficiency, as well as potentially reducing the impact of loop out recombination [25,26]. IRES are large sequences and preferentially express the upstream protein at a 10-fold higher level of expression than the downstream protein, which explains their decreasing popularity [27]. The 2A sequence functions by causing a ribosome skip that cleaves between the terminal glycine and the beginning of the final protein resulting in two separate polypeptides [[28], [29], [30]]. 2A sequences have been used in *P. pastoris* for the production of complex multi-enzyme pathways [31] as well as for the production of antibodies [32]. The original 2A sequence was discovered in foot-and-mouth disease virus (FMDV), F2A, in 1991 [33]. In addition, there are the *Thosea asigna* virus sequence (T2A) and the porcine teschovirus-1 sequence (P2A) [24]. The P2A has been used for the production of antibodies in a streamlined system outlined by Shah et al. [32]. All three variants have been analysed in *P. pastoris* [31], and Geier et al. identified that using the F2A can result in production of the fusion protein as opposed to discrete protein. Furthermore, work carried out in mouse lines indicates expression of the second protein is higher when using the T2A compared to the P2A [34].

Here we set out to increase the production of the bNAb VRC01 by exploring a combinatorial library of signal peptides, including the murine signal peptide first described in our previous paper. Furthermore, we evaluated the impact of using a 2A signal peptide to produce both chains of the antibody from a single bicistronic vector.

## 2 Materials and methods

### 2.1 Media and growth conditions

Bacterial strains were cultured in Lennox lysogeny broth (LB) medium (1% peptone au casein, 0.5% yeast extract, 0.5% NaCl) and supplemented with either 25 μg mL^−1^ Zeocin™ (Life Technologies, Carlsbad, USA) or 100 μg mL^−1^ ampicillin (Sigma Aldrich, Dorset, UK). Yeast strains were cultured in YPD medium (2% peptone au casein, 1% yeast extract, 2% dextrose). Selection was carried out using Zeocin (Thermo Fisher Scientific, Paisley, UK) and Nourseothricin (clonNAT; Jena Bioscience, Jena, Germany) at 100 μg mL^−1^ each. Expression was carried out in buffered glycerol/methanol-complex medium (BMGY/BMMY; 1% yeast extract, 2% peptone, 100 mM potassium phosphate, pH 6.0, 1.34% yeast nitrogen base, 4 × 10^−5^% d-Biotin, 1% glycerol or 0.5% methanol).

### 2.2 Strain construction

Bacterial recombinant DNA manipulation was carried out in *Escherichia coli* strain NEB 5-α (New England Biolabs, Hertfordshire, UK). The VRC01 heavy and light chains were previously cloned into either pPICZα or pJANs-1 [7]. Vectors containing 10 different signal peptides were purchased from ATUM^SM^ (Newark, California). The signal peptides include; α-amylase (AA), α-mating factor K (αK), α-mating factor KS (αKS), α-mating factor T (αT), glucoamylase (GA), insulinase (IN), invertase (IV), killer protein (KP), lysozyme (LZ) variant V11L, murine IgG1 (M) and serum albumin (SA) (Supplementary Table 1). The signal peptides were amplified using PCR and were cloned into either pPICZα-heavy or pJANs-light, replacing the α-mating factor using the Gibson DNA assembly method [35]. The PCR fragments were gel extracted using the Zymoclean™ Gel DNA Recovery kit (Zymo Research Corporation, Irvine, USA). DNA was incubated at 50 °C in equimolar concentrations of 100 ng of DNA with the ingredients of the Gibson master mix for an hour and 1 μL was transformed into NEB 5-α competent cells (New England Biolabs).

*Pichia pastoris* (syn. *Komagataella phaffi*) strain *Δku70* (CBS 12694, CBS-KNAW, Fungal Biodiversity Centre, Utretcht, The Netherlands) was used as the background strain. For cloning into *P. pastoris* 5–10 μg of plasmid DNA was linearized with PmeI at a single restriction site within the *AOX1* promoter. The vectors were transformed into cells by electroporation according to recommendations in the *Pichia* Expression manual (Thermo Fisher Scientific) and grown for 3–5 days at 30 °C on YPD medium containing both 100 μg mL^−1^ Zeocin (Thermo Fisher Scientific) and 100 μg mL^−1^ Nourseothricin (Jena Bioscience) to select for double transformants. For nomenclature purposes the heavy chain will be denoted as (H) and light chain as (L) following the signal peptide, i.e. αKH-GAL would be the combination of the α-mating factor K signal peptide fused to the heavy chain and the glucoamylase signal peptide fused to the light chain.

For the bicistronic vectors the T2A and light chain was amplified from r_αA-VRC01 and integrated into the pPICZ-heavy vector, in the orientation of light-T2A-heavy [36]. After transformation into *P. pastoris* selection was carried out on YPD plates containing 100 μg mL^−1^ Zeocin (Thermo Fisher Scientific).

### 2.3 Copy number analysis

Genomic DNA was extracted using the DNeasy^®^ Plant Mini Prep Kit (Qiagen, Crawley, UK), quantified by Nanodrop™ (Thermo Fisher Scientific) and normalized to 0.5 ng μL^−1^ using distilled H_2_O. Quantitative PCR was run on genomic DNA using KICStart^®^ SYBR^®^ Green qPCR ReadyMix™ (Sigma Aldrich) in an Eppendorf Mastercycler^®^ ep realplex quantitative cycler (Eppendorf UK Ltd, Histon, UK). The known 1 copy clone α-1 was used as a reference strain and all strains were compared accordingly [7]. Data was analysed using the Pfaffl method, based on ΔΔ-Ct [37,38] and normalized to *ACT1* as the housekeeping gene. Primers for the heavy chain were AAT CAC AAG CCC AGC AAC AC and GGG CAT GTG TGA GTT TTG TCA C resulting in a 74 bp amplicon. Primers for the light chain were TGT CTT CAT CTT CCC GCC ATC and ATT CAG CAG GCA CAC AAC AG resulting in a 70 bp amplicon. Primers for *ACT1* were GCT TTG TTC CAC CCA TCT GT and TGC ATA CGC TCA GCA ATA CC resulting in a 163 bp amplicon. Cycling conditions were 95 °C for 5 min followed by 40 cycles of 95 °C for 5 s, 58 °C for 15 s and 72 °C for 10 s with a melting curve afterwards to ensure a single product was being measured. Error bars are representative of the Gaussian error propagation of the standard deviation of s[untreated control], s[treated control], s[untreated sample] and s[treated sample] [39].

### 2.4 Protein expression

Expression screening in *P. pastoris* was performed in 24 deep-well plates in 3 mL of medium and sealed with Breathe-Easy^®^ sealing membrane (Sigma Aldrich). Larger scale expression in *P. pastoris* was performed in 250 mL glass baffled flasks in 25 mL of BMGY medium. Cells were incubated at 30 °C, 216 rpm for 24 h in BMGY to allow growth before being centrifuged at 4000 rpm for 5 min. The supernatant was removed and the medium replaced with BMMY to induce expression. Cultures were left to express at 20 °C, 216 rpm for 72 h before being harvested. Every 24 h the culture was supplemented with 0.5% (v/v) methanol.

### 2.5 Western blot analysis

Sodium dodecyl sulfate polyacrylamide gel electrophoresis (SDS–PAGE) was performed using 12% Tris-HCl SDS-PAGE gels. *P. pastoris* culture supernatants were denatured by boiling for 5 min in reducing SDS sample buffer (0.0625 M Tris–HCl, pH 6.8, 2.3% (w/v) SDS, 10% (w/v) glycerol and 0.01% Bromophenol blue, 5% (v/v) β-mercaptoethanol). To assess intracellular protein the *P. pastoris* pellet was treated as previously described [40]. Molecular weight was estimated by using a prestained protein ladder (10–170 kDa, Thermo Fisher Scientific). Transfer to an Immobilon^®^-FL PVDF membrane (Millpore (U.K) Ltd, Herfordshire, UK) was performed using a Novex^®^ semi-dry blotter (Thermo Fisher Scientific). VRC01 antibody was detected by using a 1:5000 dilution of an AP-conjugated Rabbit Anti-Human IgG H&L (Abram, Cambridge, UK). Bands were developed using the alkaline phosphate substrate BCIP/NBT kit (Thermo Fisher Scientific).

### 2.6 Enzyme-linked immunosorbent assay (ELISA)

ELISA was performed on neat supernatant cultures from flask expression. For the total Ig ELISA, Nunc Maxisorp (Thermo Fisher Scientific) high-binding 96-well microplates were coated with 100 μL anti-human Kappa and Lambda light chain mix (Southern Biotech, Birmingham, US) in PBS at 4 °C overnight. Wells were washed with 0.05% (v/v) Tween in PBS four times, and subsequently blocked with 200 μL 0.05% (v/v) Tween and 1% (v/v) Bovine Serum Albumin (BSA) in PBS (assay buffer) for 1 h at 37 °C. To generate a standard curve 50 μL standard antibody in 5-fold serial dilution was added to triplicate wells, with a starting concentration of 1000 ng mL^−1^. The plates were then incubated for 1 h at 37 °C, washed, and 100 μL detection antibody added (anti-human IgG HRP; Southern Biotech), diluted to 1:10,000 in assay buffer. After 1 h incubation at 37 °C wells were washed a further 4 times. 50 μL TMB (KPL Inc, Milford, USA) was added and incubated for 5 min 50 μL of Stop solution (KPL Inc) was added and the absorbance measured at 450 nm immediately. Some ELISAs included a biotin amplification step where the detection antibody used was an anti-human IgG Biotin labelled (Southern Biotech), diluted to 1:10,000 in assay buffer, followed by a wash step and then incubation with a streptavidin-HRP conjugate (R&D Systems), diluted 1:200 in assay buffer, before washing and development of the signal with TMB as detailed above. To determine antigen-specific binding wells were coated with 50 μL CN54-gp140 antigen at 1 μg mL^−1^ in PBS at 4 °C in place of the IgG capture antibodies. The antibody concentration reported by the gp140 ELISA method is lower than that of the total antibody method due to different binding affinities to the plate bound antigen. Yields of VRC01 for shake flasks were recorded previously [7].

## 3 Results and discussion

### 3.1 Expression of VRC01 using the paired signal peptide

**Fig. 1:**
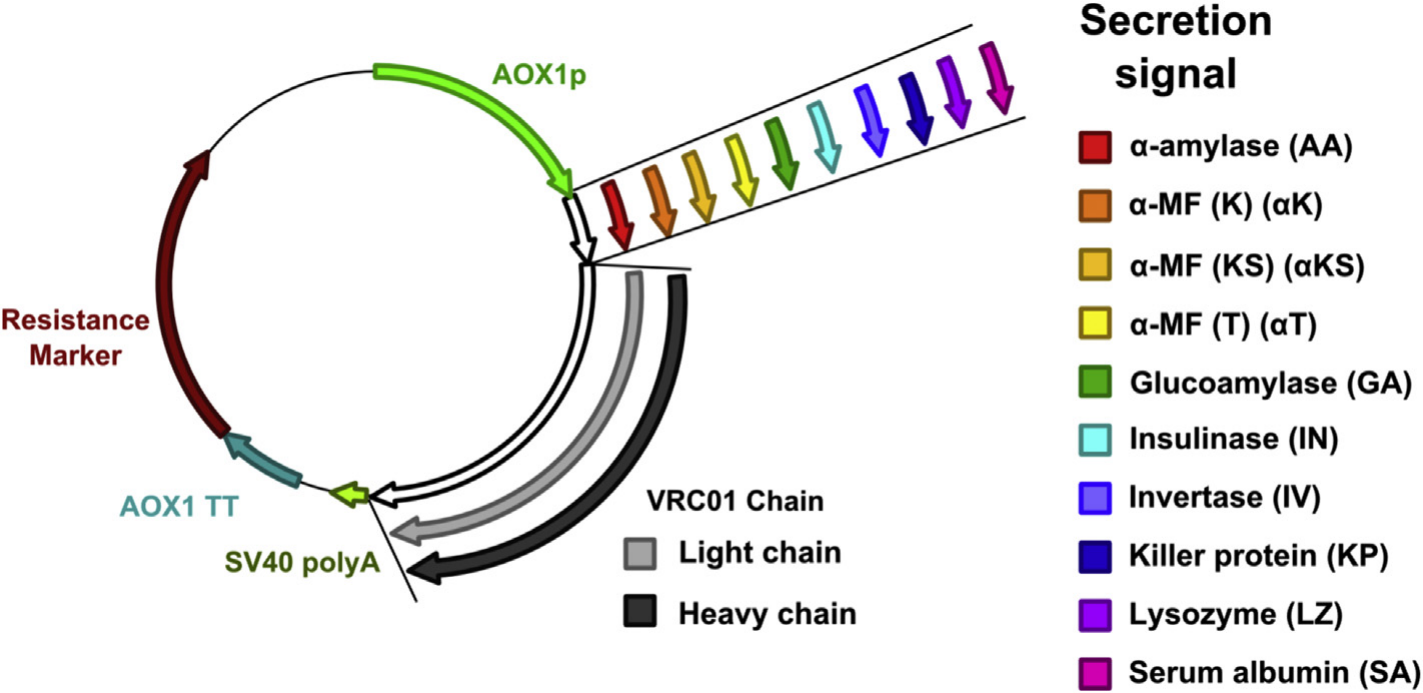
**Graphical representation of vectors.** Twenty vectors were generated containing a different signal peptide combined with the light or the heavy chain.

The signal peptides from the ATUM^SM^ expression library vectors were amplified and cloned into the existing pPICZ-heavy or pJAN-light vectors [7]. This created 20 vectors, containing the 10 signal peptides with either the light or heavy chains in addition to the original vectors containing the murine signal peptide (Fig. 1). Of the new 10 vectors, each vector pair containing the same signal peptide (e.g. SAH and SAL) on the heavy and light chain was transformed in Δ*ku70* with selection on both 100 μg mL^−1^ Zeocin and 100 μg mL^−1^ cloNAT. This background strain was chosen as it prevents off-target recombination, and as both vectors integrate into the same locus ensures correct integration [41]. The presence of both the heavy and light chain sequences was confirmed by colony PCR as previously described (data not shown) [7].

**Fig. 2:**
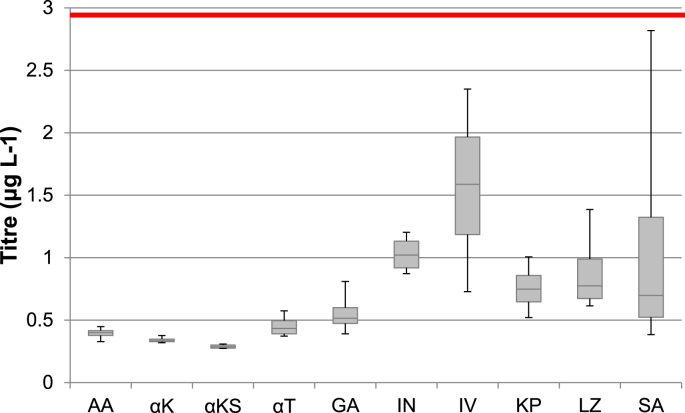
**Concentration of total antibody by ELISA.** Antibody concentrations were calculated of total IgG1 antibody. Four clones of each condition were expressed for 72 h in BMMY and are represented in box plot format. The red line indicates the highest yield achieved from M2 [7] at 2.95 μg L^−1^. The error bars are representative of the standard deviation from four independent clones.

**Fig. 3:**
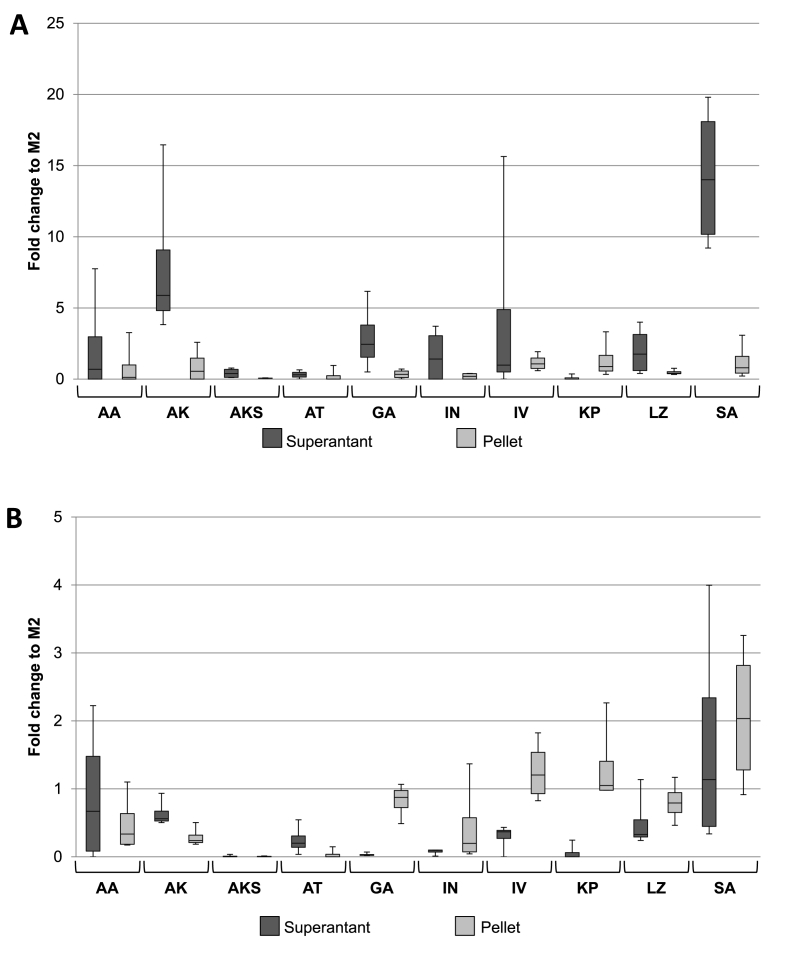
**Relative expression of heavy and light chains compared to M2.** From Western blots the densitometry of bands was calculated in relation to secreted M2 heavy and light chain. Ratios of both the supernatant and the pellet were calculated and plotted on the same graph. Box plots are representative of four independent clones from each signal peptide. **A)** Light chain **B)** Heavy chain.

Emulating the screening process that is used in industry, we randomly selected four independent clones to test for expression levels [42,43]. Clones were expressed in microtiter plates and the expression was determined by ELISA (Fig. 2) and Western blot (Fig. 3 and Supplementary Fig. 1). In our original paper, the most highly expressed strain was M2, which in small scale produced 2.95 μg L^−1^, which is represented on Fig. 2 with the red line. None of the strains with new signal peptides outperformed this strain in small scale expression (Fig. 2). However, there was noticeable variation in expression of the heavy and light chains when culture supernatants were analyzed by Western blot, as indicated in the representative Western blots shown in Supplementary Fig. 1. For example, using the GA signal peptide resulted in an abundance of light chain, but limited amounts of heavy chain, with most being retained in the pellet (Supplementary Fig. 1). On the other hand, using the SA signal peptide resulted in higher levels of expression of the heavy chain than in GA, although there is still evidence that the heavy chain was not fully secreted (Supplementary Fig. 1). The ratio of light to heavy chain is more balanced when using the SA signal peptide, and this is represented in the ELISA results (Fig. 2) where there is increased signal using the SA signal peptide compared to the GA signal peptide.

Western blot heavy and light chain bands were measured by densitometry and calculated as a ratio to the previously published M2 strain (Fig. 3). Light chains were highly secreted in strains with the αK, GA, IV, LZ and SA signal peptides, although IV shows proportionally higher levels of light chain maintained within the pellet than the other strains. Conversely the αKS, αT and KP signal peptides resulted in very low levels of light chain secretion, with the former two showing also very little retained within the pellet (Fig. 3A). For the heavy chain, the AA, αK, LZ and SA signal peptides show the highest levels of secretion, although all also show antibody being retained within the pellet. Levels of antibody in strains using the αKS are low, but this could be due to the low levels of light chain production in these strains. αT shows higher levels of heavy chain in the supernatant than is retained in the pellet, and this implies that the low total antibody yield observed for this strain (Fig. 2) is not as a result of the heavy chain being maintained within the pellet, but lower light chain expression, suggesting that this signal peptide is not compatible with high levels of production of VRC01.

### 3.2 Expression of VRC01 using combination signal peptides

The Western blot results (Fig. 3 and Supplementary Fig. 1) facilitated selection of signal peptides for further study. It is apparent that the choice of signal peptides affects the efficiency of secretion of heavy and light chains and therefore, using a combination of different signal peptides could increase yield. To select which signal peptides to explore in our combinatorial library, we used different criteria for the heavy and light chain. It has been shown that light chain can be secreted independently of the heavy chain, but not the other way around [12,44,45]. Therefore, signal peptides from strains that showed high levels of light chain in the supernatant where chosen for further strain construction irrespective of the secretion of the heavy chain. For the heavy chain, signal peptides were chosen based on either a high level of expression in the supernatant from the ELISA data (e.g. SA, LZ) or those that showed low levels of expression of the light chain and evidence of heavy chain accumulation in the cell pellet (e.g. AA, KP) since the antibody secretion in these strains could have been negatively impacted by low availability of the light chain. As a result, the following signal peptides were chosen to be fused with the heavy chain: AA, αK, αKS, IV, KP, LZ and SA. For the light chain the αK, GA, LZ and SA signal peptides were chosen. Furthermore, as the murine signal peptide resulted in the highest levels of secretion in our previous study, this was also used for both the heavy and the light chains. Therefore, we constructed a combinatorial library of 36 different sets of strains by transforming each combination of heavy and light chain vector into *P. pastoris*.

**Fig. 4:**
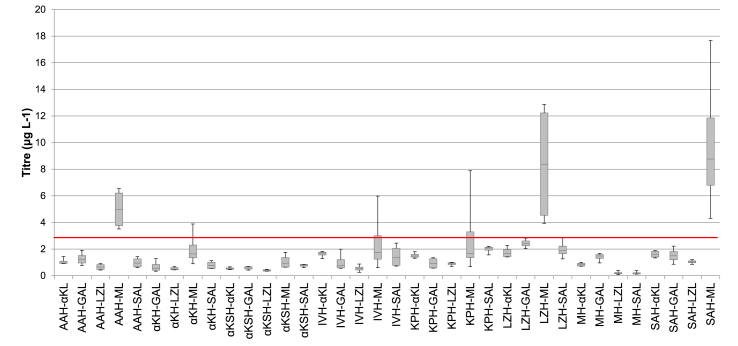
**Concentration of total antibody by ELISA for combination strains.** Antibody concentrations were calculated of total IgG1 antibody by ELISA. Four clones of each condition were expressed for 72 h in BMMY and are represented in box plot format. The red line indicates the highest yield achieved from M2 [7] at 2.95 μg L^−1^. The error bars are representative of the standard deviation from four independent clones. (For interpretation of the references to colour in this figure legend, the reader is referred to the Web version of this article.)

Four colonies from each combination were selected and antibody was expressed in microtiter plates as previously described. Antibody yield was analysed by ELISA (Fig. 4) and Western blots (data not shown). Compared to the previously reported strain that used the murine secretion signal for both heavy and light chain (indicated by the red line), there were strains with higher yield, unlike when using identical signal peptides from the library for both the heavy and the light chains. Interestingly, the murine signal peptide appeared to be the best fusion partner for the light chain in all cases. As we have analysed multiple clones in order to account for clonal variation [42,43] it was of note that all clones of AAH-ML, LZH-ML and SAH-ML combination outperformed the M2 strain in small scale expression, suggesting that these signal peptides result in efficient processing of the antibody for secretion. The best secreting clones of AAH-ML, LZH-ML and SAH-ML produced 1.5, 5 and 7 fold higher titers than M2, respectively.

**Fig. 5:**
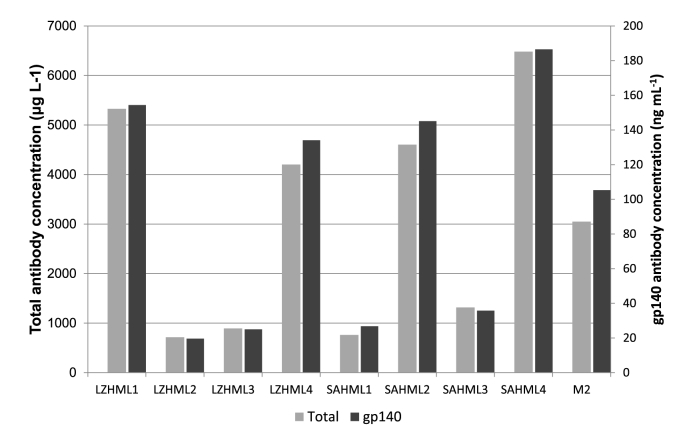
**Concentration of total and gp140 specific antibody from large scale expression of LZHML and SAHML.** Antibody concentrations were calculated for total IgG1 and gp140 specific antibody. Samples were expressed in BMMY for 72 h in 25 mL cultures. M2 is representative of previous results reported from large scale expression [7].

The LZH-ML and SAH-ML strains had the highest antibody expression levels overall. Therefore, we scaled up expression of these strains to shake flasks and compared the titers of total and gp140 specific antibody to those from M2 (Fig. 5). Despite the small-scale expression results, not all of the clones outcompete M2 in antibody yield in shake flask expression. It has been reported previously that reproducible scale up is difficult to achieve in *P. pastoris*, often due to the high oxygen levels required, which can result in cell death [46]. Nonetheless compared to M2, two clones of each of the strains produced higher total and gp140 specific antibody concentrations (LZHML1, LZHML4, SAHML2 and SAHML4).

As our initial selection of clones was random, we undertook an investigation to understand if the differences in yield observed from different strains were solely a result of differences in gene copy number [43]. In the industrial selection of clones, companies will often screen thousands of colonies based on titer alone, and only assess integration sites and copy number after a good candidate has been identified [47]. Therefore, from our best secreting clones we analysed the copy number of the four LZHML and SAHML strains to ensure that the differences we had measured for titer were not purely as a result of copy number differences. We had previously determined that the M2 strain has two copies of both of the heavy and the light chain. Interestingly, the lower expressing clones of LZHML and SAHML had higher copy numbers, ranging from 4 to 8 copies of the heavy chain and 3 to 6 copies of the light chain (Supplementary Fig. 2). This is consistent with previous findings that implied the benefits of increasing gene dosage plateau at a maximum value where secretion saturation can occur, resulting in detrimental effects on yield on further increase in copy number [26]. In fact, there was a negative correlation between the number of heavy chain copies and the level of expression (r = 0.715), which was less pronounced for the light chain correlation (r = 0.5951). This is supported by the findings that the heavy chain cannot be secreted without the light chain; therefore the heavy chain remains the limiting factor for the concentration of intact antibody [12,44,45].

From the best secreting strains LZHML1, SAHML2 and SAHML4 had one copy of heavy chain and two copies of light chain whilst LZHML4 had two copies of each the heavy and the light chains (comparable to the M2 strain analysed). This indicates that the variation observed between the levels of expression using different signal peptides is not due to differences in copy number, but results from the different signal peptides.

Whilst there is no significant difference when comparing all of the strains, SAHML4 had a total expression of 6.5 mg L^−1^ and 187 μg L^−1^ of gp140 specific antibody. This is a 2.13 and 1.77 fold increase respectively compared to M2, which was the highest secretor found from our previous experiments. Even though our yields are still comparatively low compared to those reported for other types of proteins expressed in *P. pastoris* [10], they are on par with that previously reported for expression of other antibodies. For example, Shah et al. reported yields of 100–500 μg L^−1^ for antibody production in microtiter plates and scFvs titers of up to 3.5 mg L^−1^ were reported in shake flasks (in a mutS strain KM71) [32,48,49]. Optimising growth conditions and using bioreactors may result in further improved yield. Furthermore, as the VRC01 gene used was not codon optimized, this may have limited productivity, although codon optimization does not always result in improved yields [50].

### 3.3 Expression of VRC01 using the T2A signal peptide

A recent investigation into creating an automated pipeline for the production of HIV-specific monoclonal antibodies in *P. pastoris* utilized a 2A peptide sequence to generate a single vector to express both the heavy and the light chains [32]. We decided to generate bicistronic vectors for the MHML, LZHML and SAHML combinations as these gave the highest yields when utilising separate plasmids. As the T2A sequence from *Thosea asigna* virus was shown to produce discrete proteins in a previous investigation and results in higher expression of the second protein, this peptide sequence was used as a linker between the light and heavy chains [31,34]. A single bicistronic vector should reduce the burden on *P. pastoris* and increase transformation efficiency [25,51]. It has previously been demonstrated that an excess of light chain can promote folding and increase antibody stability; therefore the light chain was placed upstream of the T2A signal peptide [32,52].

**Fig. 6:**
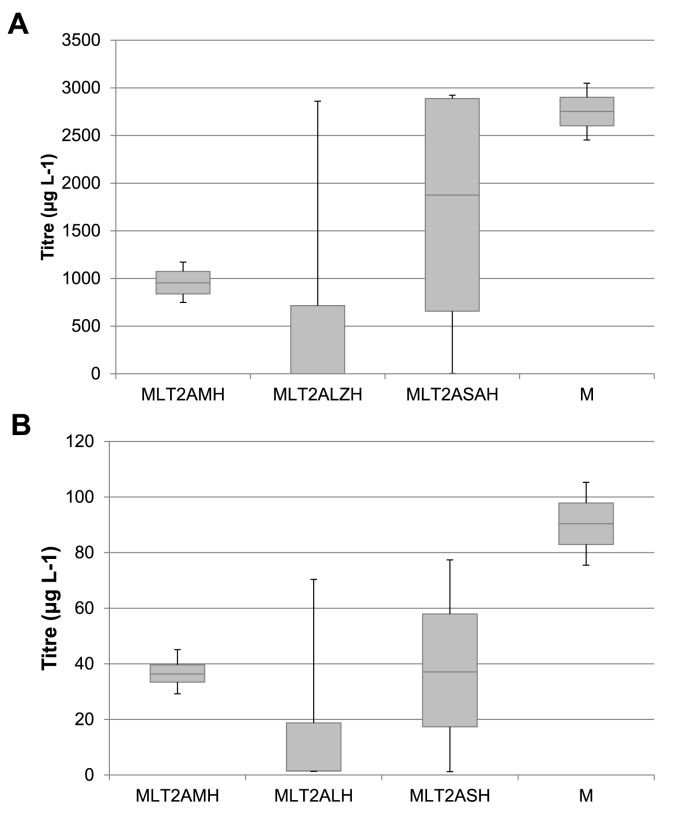
**Concentration of total and gp140 specific antibody of MLT2ALZH, MLT2SAH and MLT2AMH.** Antibody concentrations were calculated of total IgG1 and gp140 specific antibody. Samples were expressed in BMMY for 72 h in 25 mL cultures. M is representative of previous results reported from large scale expression [7]. **A)** Total antibody concentration. **B)** gp140 specific antibody concentration. The error bars are representative of the standard deviation from four independent clones.

Initially, twenty clones from each of the transformations were expressed in microtiter plates and the yield observed via dot blot (data not shown). From the dot blot results, we selected four of the highest expressing clones, which were expressed in shake flasks for 72 h in the methanol containing medium BMMY (Fig. 6). Despite the reduced burden, no clones showed higher expression than the M2 strain originally described. The MLT2AMH strains had a mean total antibody expression of 958 μg L^−1^ and a mean of 36.8 μg L^−1^ of gp140 specific antibody, both over a third lower than the M2 strain with the same signal peptides. The highest yield came from the MLT2SAH strains; however, the mean expression was still lower at 1.7 mg L^−1^ and 38.2 μg L^−1^ for total antibody and gp140 specific antibody concentrations respectively. The highest individual expressing strain was MLT2ASAH1 at 2.9 mg L^−1^ of total antibody, although for gp140 specific antibody this was MLT2ASAH4 at 77.7 μg L^−1^. We previously reported that the signal from the gp140-specific ELISA was between 4 and 5% that of the total antibody ELISA for antibody expressed in mammalian 293T cells as well as *P. pastoris* [7]. This difference is due to different binding affinities of the antibody to the plate bound antigen, in contrast to the much higher affinity capture ELISA used to detect total IgG. The plate bound antigen may be distorted due to electrostatic stresses, leading to a reduction in the ability of the antibody to bind. However, here we see variations in this percentage depending on the signal peptide used, with ranges between 1.76% and 4.3%. Since some strains show percentages in the same range as our previous measurements, this disparity is unlikely to be due to incomplete processing of the 2A sequence and is perhaps more likely to be signal peptide related (Supplementary Fig. 3).

It would appear from our findings that the use of a peptide cleavage signal was not effective for increasing the expression of the VRC01 antibody. Further investigations are required to determine if this is specific for T2A or whether other 2A peptides may result in higher yields, or whether placing the heavy chain upstream of the light chain results in increased production.

## 4 Conclusions

Through the combinatorial exploration of signal peptides, we have increased the expression of the bNAb VRC01 in *P. pastoris* by 2–7 fold with yields of up to 6.5 mg L^−1^ in shake flask expression. This was through the combination of different signal peptides on both the heavy and the light chain. We have determined that the use of the murine IgG1 signal peptide resulted in the highest yields when fused to the light chain, with greater flexibility on the signal peptide for the heavy chain. Surprisingly the use of the T2A peptide sequence resulted in lower yields than when using independent plasmids. Further investigations into the choice of a cleavage sequence would be required to determine whether this is the most efficient approach for antibody production in *P. pastoris*.

## Author contributions

RA designed and performed the experiments and helped draft the manuscript. PM performed the ELISA experiments and analysis. RS, PM and KP helped design the experiments and draft the manuscript. RS and KP conceived the study. All authors read and approved the final manuscript.

## Declarations of interest

None.

## Appendix A Supplementary data

Data Profile Data Profile

Supplementary Fig. 1 **Western blot of GA and SA strains.** Representative Western blots indicating heavy and light chains. Samples were run on a denaturing gel, using a secondary rabbit anti-human IgG heavy and light antibody. A) glucoamylase (GA); B) serum albumin (SA). Supplementary Fig. 1

Supplementary Fig. 2 **Copy number of heavy and light chains of LZHML and SAHML.** Copy number was determined by qPCR using the Pfaffl method against a known one copy clone. Error bars are calculated using the Gaussian error propagation of the standard deviation of s[untreated control], s[treated control], s[untreated sample] and s[treated sample]. Supplementary Fig. 2

Supplementary Fig. 3 **Western blot of T2A bicistronic strains**. Expression from large scale cultures were run on a denaturing gel, using a secondary rabbit anti-human IgG heavy and light antibody. **L)** Ladder, **1)** MLT2AMH-1, **2)** MLT2AMH-2, **3)** MLT2AMH-3, **4)** MLT2AMLH-4, **5)** MLT2ALZH-1, **6)** MLT2ALZH-2, **7)** MLT2ALZH-3, **8),** MLT2ALZH-4, **9)** MLT2ASAH-1, **10)** MLT2ASAH-2, **11)** MLT2ASAH-3, **12)** MLT2ASAH-4, **13)** M2. Supplementary Fig. 3
