# Supplementary figures and images for: A systematic analysis of the expression of the anti-HIV VRC01 antibody in Pichia pastoris through signal peptide optimization
Source: Protein Expr Purif. 2018 Sep;149:43–50. doi: 10.1016/j.pep.2018.03.013 (PMC5982643; doi:10.1016/j.pep.2018.03.013)

## Slide 1
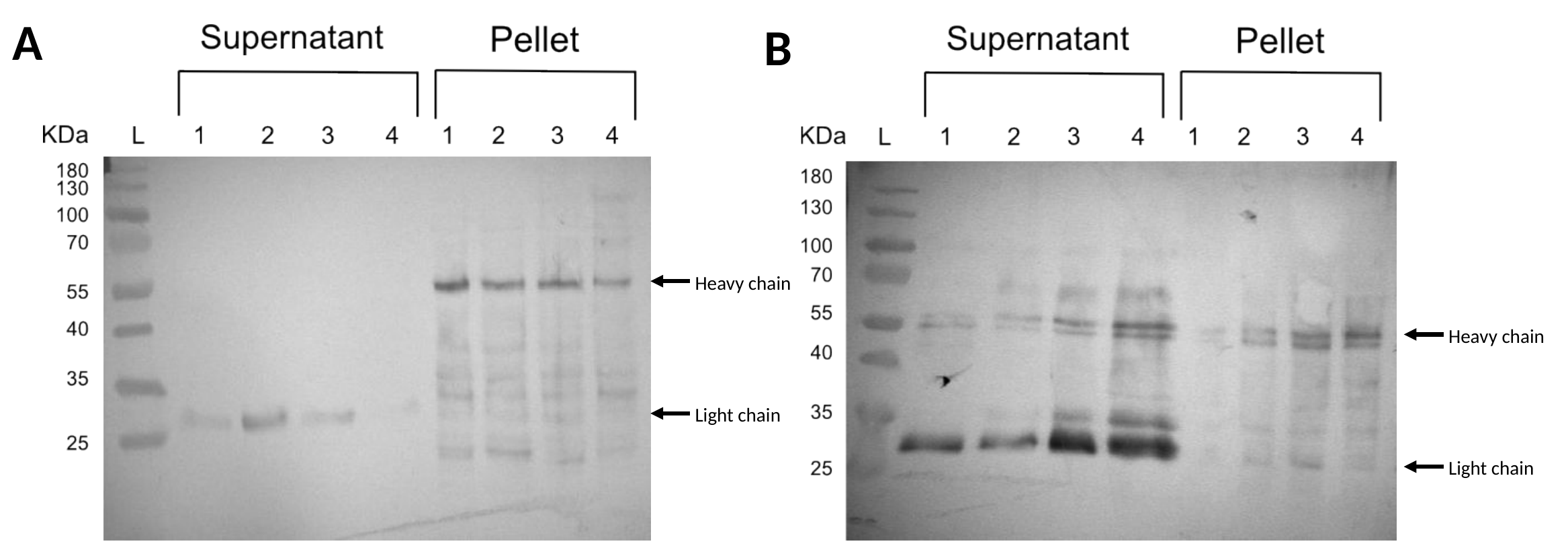

A
B
Heavy chain
Heavy chain
Light chain
Light chain

Supplement: Supplementary Fig. 1 — Western blot of GA and SA strains. Representative Western blots indicating heavy and light chains. Samples were run on a denaturing gel, using a secondary rabbit anti-human IgG heavy and light antibody. A) glucoamylase (GA); B) serum albumin (SA). [file mmc2.pptx]

## Slide 1
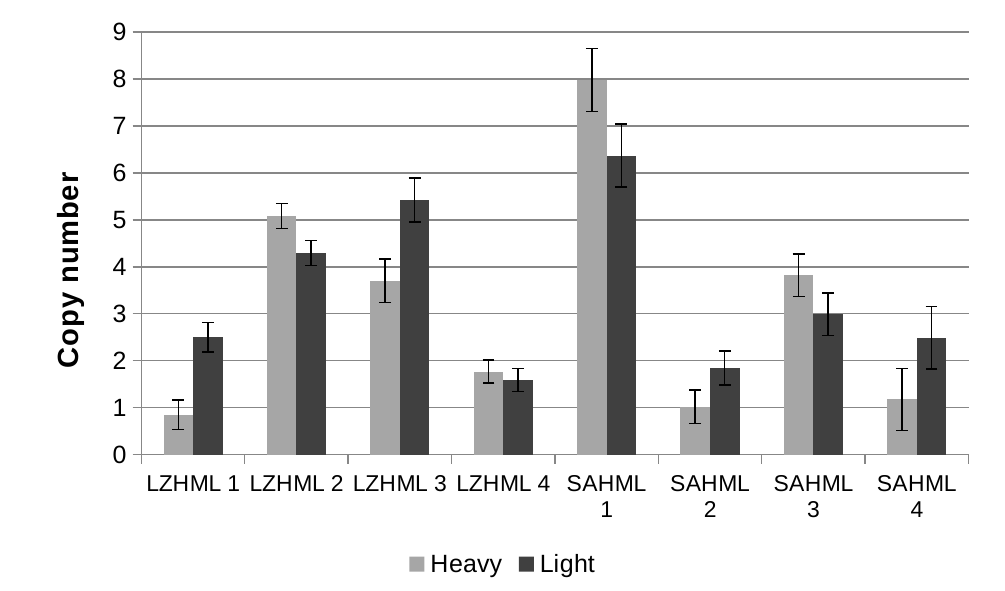

### Chart
| Category | Heavy | Light |
|---|---|---|
| LZHML 1 | 0.853116499124459 | 2.498552450238061 |
| LZHML 2 | 5.081397136401783 | 4.293096732549552 |
| LZHML 3 | 3.6982802729946513 | 5.420647284943799 |
| LZHML 4 | 1.768535975505476 | 1.5874592752091952 |
| SAHML 1 | 7.972912910762446 | 6.364137429867453 |
| SAHML 2 | 1.0175616473420448 | 1.8439328421589383 |
| SAHML 3 | 3.820992534703139 | 2.984078889365515 |
| SAHML 4 | 1.1747264570709963 | 2.4878839213057544 |

Supplement: Supplementary Fig. 2 — Copy number of heavy and light chains of LZHML and SAHML. Copy number was determined by qPCR using the Pfaffl method against a known one copy clone. Error bars are calculated using the Gaussian error propagation of the standard deviation of s[untreated control], s[treated control], s[untreated sample] and s[treated sample]. [file mmc3.pptx]

## Slide 1
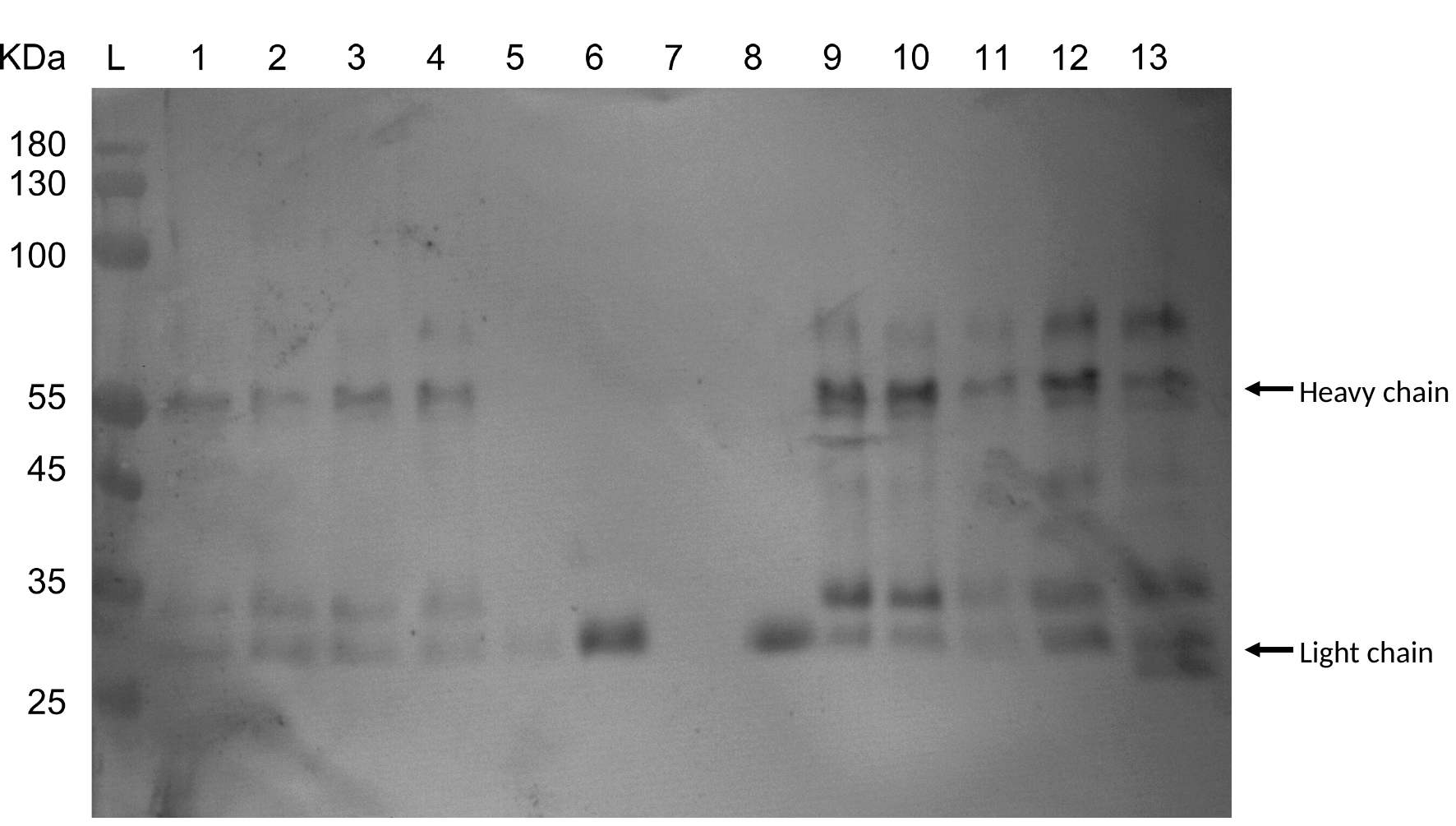

Heavy chain
Light chain

Supplement: Supplementary Fig. 3 — Western blot of T2A bicistronic strains. Expression from large scale cultures were run on a denaturing gel, using a secondary rabbit anti-human IgG heavy and light antibody. L) Ladder, 1) MLT2AMH-1, 2) MLT2AMH-2, 3) MLT2AMH-3, 4) MLT2AMLH-4, 5) MLT2ALZH-1, 6) MLT2ALZH-2, 7) MLT2ALZH-3, 8), MLT2ALZH-4, 9) MLT2ASAH-1, 10) MLT2ASAH-2, 11) MLT2ASAH-3, 12) MLT2ASAH-4, 13) M2. [file mmc4.pptx]
