# Supplementary material for: A systematic analysis of the expression of the anti-HIV VRC01 antibody in Pichia pastoris through signal peptide optimization
Source: Protein Expr Purif. 2018 Sep;149:43–50. doi: 10.1016/j.pep.2018.03.013 (PMC5982643; doi:10.1016/j.pep.2018.03.013)
Supplement: Supplementary Table 1 [file mmc5.docx]

Supplementary table 1. Nomenclature used for different signal peptides

| **Signal peptide** | **Nomenclature used** |
| --- | --- |
| α-amylase | AA |
| αMF (K) | αK |
| αMF (KS) | αKS |
| αMF (T) | αT |
| Glucoamylase | GA |
| Insulinase | IN |
| Invertase | IV |
| Killer protein | KP |
| Lysozyme | LZ |
| Murine | M |
| Serum albumin | SA |
